# Knockout of ENO1 leads to metabolism reprogramming and tumor retardation in pancreatic cancer

**DOI:** 10.3389/fonc.2023.1119886

**Published:** 2023-02-10

**Authors:** Qingru Song, Kai Zhang, Tianjiao Sun, Congcong Xu, Wei Zhao, Zhiqian Zhang

**Affiliations:** Key Laboratory of Carcinogenesis and Translational Research (Ministry of Education), Department of Cell Biology, Peking University Cancer Hospital & Institute, Beijing, China

**Keywords:** ENO1 DEGs, ENO1-knockout, pancreatic cancer (PC), tumor metabolism, tumorigenicity

## Abstract

The shift in glucose utilization from oxidative phosphorylation to glycolysis is the hallmark of tumor cells. The overexpression of ENO1, one of the key enzymes in the glycolysis process, has been identified in several cancers, however, its role in pancreatic cancer (PC) is yet unclear. This study identifies ENO1 as an indispensable factor in the progression of PC. Interestingly, ENO1-knockout could inhibit cell invasion and migration and prevent cell proliferation in pancreatic ductal adenocarcinoma (PDAC) cells (PANC-1 and MIA PaCa-2); meanwhile, tumor cell glucose uptake and lactate excretion also decreased significantly. Furthermore, ENO1-knockout reduced colony formation and tumorigenesis in both *in vitro* and *in vivo* tests. In total, after ENO1 knockout, 727 differentially expressed genes (DEGs) were identified in PDAC cells by RNA-seq. Gene Ontology enrichment analysis revealed that these DEGs are mainly associated with components such as the ‘extracellular matrix’ and ‘endoplasmic reticulum lumen’, and participate in the regulation of signal receptor activity. Kyoto Encyclopedia of Genes and Genomes pathway analysis revealed that the identified DEGs are associated with pathways, such as ‘fructose and mannose metabolism’, ‘pentose phosphate pathway, and ‘sugar metabolism for amino and nucleotide. Gene Set Enrichment Analysis showed that ENO1 knockout promoted the upregulation of oxidative phosphorylation and lipid metabolism pathways-related genes. Altogether, these results indicated that ENO1-knockout inhibited tumorigenesis by reducing cell glycolysis and activating other metabolic pathways by altering the expression of *G6PD, ALDOC, UAP1*, as well as other related metabolic genes. Concisely, ENO1, which plays a vital role in the abnormal glucose metabolism in PC, can be exploited as a target to control carcinogenesis by reducing aerobic glycolysis.

## 1 Introduction

Pancreatic cancer (PC) is a malignant tumor of the digestive system and has a high mortality rate (1). It progresses rapidly and in the lack of specific symptoms and biomarkers, its early diagnosis is challenging (2). Therefore, it is crucial to find new targets for early diagnosis and treatment of PC. The special hypoxic microenvironment involves metabolic reprogramming in pancreatic ductal adenocarcinoma (PDAC) (3). Aerobic glycolysis instead of mitochondrial oxidative phosphorylation (OXPHOS) is used for energy production, known as the “Warburg effect” (4). Glycolysis supports the vigorous growth of cancer cells by producing various substrates (5). Therefore, metabolic regulation by downregulating key glycolytic enzymes can be a novel therapy for PC.

Enolase, one of the key enzymes in glycolysis, converts 2-phosphoglycerate to phosphoenolpyruvate (6). It has 4 isoforms: ENO1 (α-enolase) is expressed in most tissues, while ENO3 (β-Enolase) is mainly expressed in muscles. Meanwhile, ENO2 (γ-Enolase) and ENO4 are mainly found in neural tissue and mouse spermatozoa, respectively (7, 8). In addition to glycolytic functions, ENO1 plays various roles in the pathophysiological environment, including cell growth, cell invasion, ischemia and hypoxia, immune tolerance, allergic reaction, metastasis,tumorigenesis and inflammatory responses, etc (9–11).

The multifunctional glycolytic enzyme ENO1 has been shown to be commonly over-expressed in various human cancers including PC (12, 13). High expression of ENO1 is positively correlated with clinical stage, lymph node metastasis, and poor prognosis in PC. ENO1 promotes pancreatic cancer cells migration and metastasis through combining with integrins and uPAR (14). *In vivo*, the monoclonal antibody that block the binding of ENO1 with plasminogen inhibits metastasis formation of PDAC cells (15). In genetically engineered mice with pancreatic cancer, ENO1 DNA vaccine elicits antitumor immune responses by decreasing numbers of myeloid-derived suppressor cells and T-regulatory cells and increasing T-helper 1 and 17 responses to prolong survival (16). ENO1 may function as a promising and clinically-relevant molecular target for immunotherapeutic strategy, particularly in pancreatic cancer (15–17).

Herein, we knocked out ENO1 in pancreatic cancer cell lines and evaluated its impact on maintaining the Warburg effect and tumor growth through biochemical and functional approaches. We show that ENO1 knockout decreased PDAC cell growth, and suppressed tumorigenesis by altering the expression of metabolic pathway-related genes to trigger the metabolic patterns shift from glycolysis to OXPHOS and other metabolic pathways.

## 2 Materials and methods

### 2.1 Cell culture

Two kinds of human PDAC cell lines, PANC-1 and MIA PaCa-2, were obtained from the American Type Culture Collection (ATCC). The cells were cultured in DMEM (Dulbecco’s Modified Eagle Medium) added with 10% fetal bovine serum (FBS), penicillin (Thermo Fisher Scientific, USA), and 100 µg/mL of streptomycin (Thermo Fisher Scientific, USA) at 37°C and 5% CO_2_.

### 2.2 Vector construction and ENO1-knockout stable cell line

The single guide RNA (sgRNA) sequences were obtained based on the *ENO1* gene sequence and cloned into the LentiCRISPR v2 plasmid using the BsmBI site and primers 5’- TCGCGGGAATCCCACTGTTG-3’(forward) and 5’-CAACAGTGGGATTCCCGCGA-3’ (reverse). For ENO1 knockout cells, we generated the recombinant lentiviruses using ViraPower Packaging Mix (Invitrogen, CA). The two knockout cell lines were obtained by infecting with lentiviruses for 48 h, followed by 2mg/L puromycin (Thermo Fisher Scientific, USA) selection for 14 days. The selected cells were subcultured to obtain the monoclonal cells. Eventually, ENO1 knockouts were confirmed by detecting ENO1 protein deficiency in corresponding cells using Western blotting.

### 2.3 Western blotting

Total cell proteins were extracted after cell lysis in RIPA lysis buffer (Solarbio, R0010). The protein-transferred PVDF membrane (Millipore, PVH00010) was incubated in 5% non-fat milk at 25°C for 1 h, followed by washing in 1×TBST. Next, the membrane was incubated with ENO1 antibodies (Sigma-Aldrich, WH0002023M1) for 1 h. After washing in TBST, the membrane was incubated with HRP-conjugated goat anti-mouse antibodies (ZSGB-BIO, ZB-2305, 1:100000) for 1 hr at room temperature. Immobilon™ Western Chemiluminescent HRP substrate (Millipore, WBKLS0500) was used to illuminate the protein bands.

### 2.4 Cell Counting Kit-8 (CCK-8) proliferation assay

Cells (5×10^3^ cells/well) were seeded in 96-well plates. The cell viability was detected every 24 h for 4 days using the CCK-8 Kit (Dojindo, CK04) according to the manufacturer’s instructions. Briefly, 10% CCK-8 solution was added to each well for 2 h, and then sample optical density (OD) was recorded at 485 nm using a microplate reader (BMG LABTECH) to determine the cell proliferation rate.

### 2.5 Colony formation assay

Cancer cells (1000 cells/well) were seeded into 6-well plates. After 2 to 3 weeks, colonies were immobilized with 4% paraformaldehyde (PFA) for 15 min. Then, the cells were stained with 0.1% crystal violet (CV) for 15 min. The stained cells were photographed with a Canon digital camera EOS M50 and counted by ImageJ software after washing. Colony formation rate is determined by counting the colonies numbers formed per 100 cells.

### 2.6 Transwell assay

Cells resuspended in DMEM without FBS were seeded into the upper chamber of the transwell chamber (Corning, CLS3422) at a cell density of 4×10^4^ cells/well. Then, 800 μL DMEM supplemented with 20% FBS was added to the well under the chamber. The cells adhering to the upper layer of the chamber were swabbed after 24 h and then immobilized in 4% PFA for 15 min. Cells were stained with CV and then photographed and counted in four randomly selected fields under a stereomicroscope (Olympus, Japan). For the invasion assay, Matrigel (Corning, 356237) was coated on the 8 μm pores polycarbonate membrane of the transwell chamber. 4×10^4^ cells were planted into each well and cultured for 48 h and the cell migration was estimated. The migration and invasion capacity was assessed by the number of cells that migrated and stained by crystal violet in a photograph. Each experiment was repeated thrice.

### 2.7 Glucose consumption and lactate production

Cells were seeded into 6-well plates and the DMEM medium was changed every 12 h. 500 μl of the medium was collected at 0 and 8 h of incubation to measure the initial and final concentrations of glucose and lactate, respectively, using a Silman M900 bioprocess biochemistry analyzer. Finally, the cells were digested with 0.25% trypsin solution (Thermo Fisher Scientific, USA) and counted through the countess 3 automated cell counter (Thermo Fisher Scientific, USA). The lactate and glucose levels were normalized based on cell count.

### 2.8 Soft agar assay

600 μl of 0.6% agar (Sigma, A1296) was added to each well of a 24-well plate. After the 0.6% agar solidified, the digested cells mixed with 0.3% agar were inoculated into the same at 600 μl/well; each well contained 1000 cells. The colonies that were >50 μm in diameter were photographed and counted under a stereomicroscope (Olympus, Japan) after three weeks.

### 2.9 Tumorigenicity assay in NOG mice

8-week-old male NOD-SCID IL-2 receptor gamma null (NOG) mice were used to access the tumorigenesis role of ENO1. The mice were divided into two groups (control and test groups) with 6 mice each. 2×10^6^ cells were subcutaneously transplanted into the two sides of the NOG mice. The corresponding tumor sizes in mice were recorded every 5 days. The tumor volume was calculated using the formula V =L*W^2^*0.5, where L and W represent the largest and the smallest diameters, respectively. After 5-6 weeks, the mice were sacrificed for tumor collection. All animal protocols were approved by the Peking University Cancer Hospital Animal Care and Use Committee, China.

### 2.10 RNA-sequencing

The cells were lysed with TRIzol reagent (Invitrogen,15596018); the library construction and RNA-sequencing were conducted by Novogene Co., Ltd (Tianjin, China). The raw sequencing data have been deposited in the Genome Sequence Archive (GSA) database under accession number HRA001089. All of the data are also available as the sequence read archive (SRA) format in the National Center for Biotechnology Information (NCBI) with the accession number of SRP414959. The gene expression differences were analyzed using the DEseq2 software (1.20.0). Data with p-value <0.05 after adjustment were considered significantly different. The Gene Ontology (GO) and KEGG (Kyoto Encyclopedia of Genes and Genomes) pathway enrichment analyses of differentially expressed genes (DEGs) were accomplished by clusterProfiler (3.4.4); GO terms with a p-value <0.05 were considered significantly enriched. Gene set enrichment analysis (GSEA) results were visualized by Omicshare Online tools.

### 2.11 Quantitative real-time PCR (q-RT-PCR)

Total cell RNA was extracted using the RNeasy^®^ Mini Kit (QIAGEN,74104) as previously described (18). 2 μg of total RNA, Oligo-(dT)_15_, dNTPs, and MMLV reverse transcriptase (Invitrogen, 28025-021) were mixed for cDNAs synthesis. qRT-PCR was performed using the Biosystems 7500 real-time PCR system. The gene expressions were estimated by the 2^−ΔΔCt^ method. The used primers are listed as follows:

GPX7:5′-CGACTTCAAGGCGGTCAACATC-3′ (forward), 5′-TCGGTAGTGCTGGTCTGTGAAG-3′ (reverse); RGN:5′-GGAGGAAGTGTCCAACTCTCTG-3′ (forward), 5′-CAATGGTGGCAACATAGCCTCC-3′ (reverse); GMPPA:5′-GGACAGTGAGAGCCTCTTCAAG-3′ (forward), 5′-TCGAGTTCAGGATGAGCACCTC-3′ (reverse); G6PD:5′-CTGTTCCGTGAGGACCAGATCT-3′ (forward), 5′-TGAAGGTGAGGATAACGCAGGC-3′(reverse); GFPT2:5′-GCTCATCGTGATTGGCTGTGGA-3′ (forward), 5′-CAACCATCACAGGAAGCTCAGTC-3′ (reverse); MGST1:5′-GCCAATCCAGAAGACTGTGTAGC-3′ (forward), 5′-AGGAGGCCAATTCCAAGAAATGG-3′ (reverse); GSTM4:5′-TGGAGAACCAGGCTATGGACGT-3′ (forward),5′-CCAGGAACTGTGAGAAGTGCTG-3′ (reverse); GAPDH:5′-GACCCCTTCATTGACCTCAAC-3′ (forward), 5′-CTTCTCCATGGTGGTGAAGA-3′ (reverse).

### 2.12 TIMER and GEPIA based gene expression and prognostic value analysis of ENO1 in human cancers

The TIMER (https://cistrome.shinyapps.io/timer/) and GEPIA databases (http://gepia2.cancer-pku.cn/#analysis) were used to analyze the difference in ENO1 expression levels between the human cancers and paired normal tissue. ENO1 expression levels (Log_2_TPM) are displayed using box plots, with statistical significance of differential expression evaluated using the Wilcoxon test in the TIMER 2.0 version. We use log2 (TPM + 1) for log-scale and match TCGA normal and GTEx data in GEPIA databases analysis. Furthermore, The Kaplan-Meier Plotter database (http://kmplot.com/analysis/) was used to find the prognostic value of ENO1 expression level in human cancers. We choose mRNA (RNA-seq) to start KM Plotter for pan-cancer. Patients are splited by selecting best cutoff value automatically.

### 2.13 Statistical analysis

Data were analyzed by GraphPad Prism 8. The data differences were assessed by t-test and those with p-value <0.05 were considered statistically significant.

## 3 Results

### 3.1 ENO1 is significantly upregulated in human pancreatic cancer and other cancer tissues

**Figure 1:**
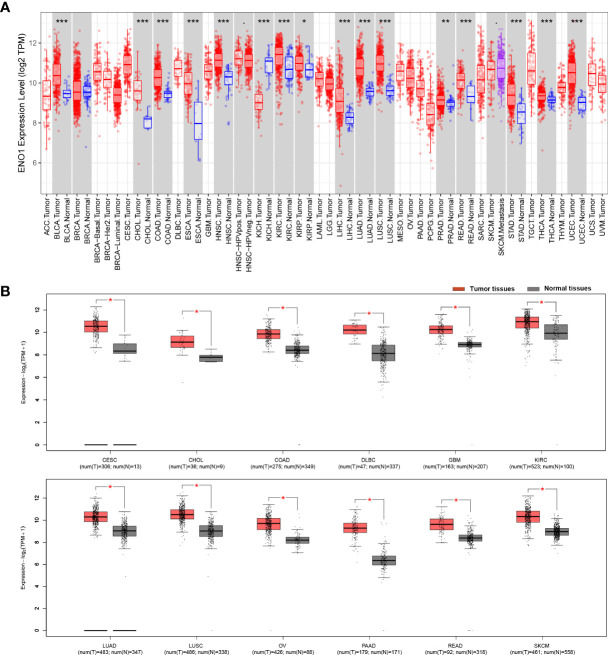
ENO1 expression is mostly upregulated different cancer types. ENO1 expression levels in different cancers and paired normal tissue were screened from the TCGA database using **(A)** TIMER (*P < 0.05, **P < 0.01, ***P < 0.001) and **(B)** GEPIA. T, tumor tissues; N, normal tissues.

The results from the TIMER database showed that ENO1 expression was significantly higher in most human cancers tissue compared to adjacent normal tissue, such as in BLCA (bladder urothelial carcinoma), BRCA (breast invasive carcinoma), CHOL (cholangiocarcinoma), COAD (colon adenocarcinoma), ESCA (esophageal carcinoma), HNSC (head and neck cancer), KIRC (kidney renal papillary cell carcinoma), KIRP (kidney renal papillary carcinoma), LIHC (liver hepatocellular carcinoma), LUAD (lung adenocarcinoma), LUSC (lung squamous cell carcinoma), PRAD (prostate adenocarcinoma), READ (rectum adenocarcinoma), STAD (stomach adenocarcinoma), THCA (thyroid carcinoma) and UCEC (uterine corpus endometrial carcinoma). Notably, ENO1 expression was significantly lower only in KICH (kidney chromophobe) **(** **Figure 1A** **)**. Similarly, GEPIA data also showed that ENO1 expression was high in most cancer types, which was consistent with TIMER analysis. The expression of ENO1 is significantly greater in PAAD (pancreatic adenocarcinoma) tumor tissues (T) than in normal tissues (N) with the highest Log_2_FC value (**Figure 1B** and **Supplementary Figure 1A** **)**. Altogether, these results suggested that ENO1 may promote the occurrence of various cancers including pancreatic cancer.

### 3.2 ENO1 is a prognostic biomarker in various cancers including pancreatic cancer

**Figure 2:**
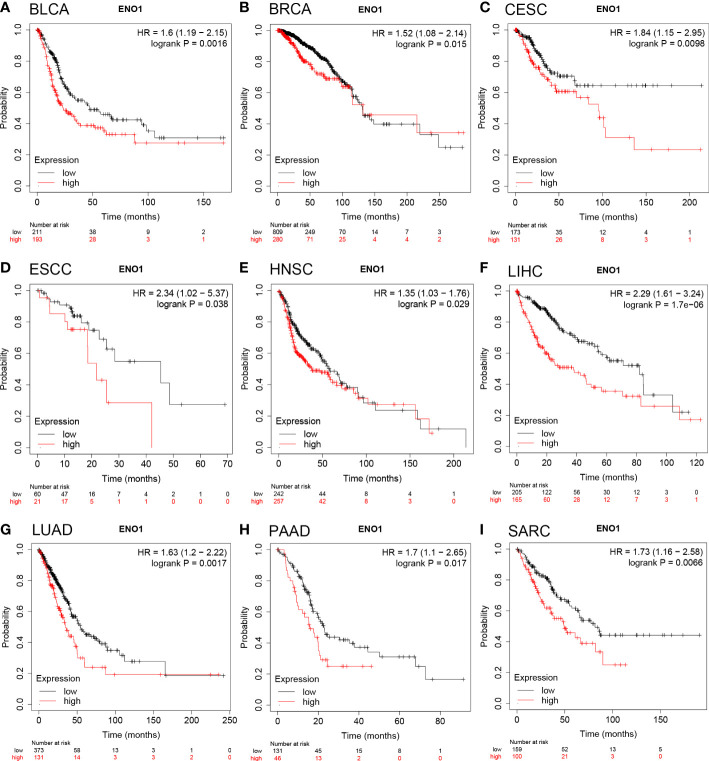
Overall survival of cancer patients was associated with high expression levels of ENO1 as analyzed by the Kaplan-Meier plotter database. **(A–I)** High ENO1 expression levels were related to worse overall survival in BLCA (n = 404), BRCA (n = 1089), CESC (n = 304), ESCC (n = 81), HNSC (n = 499), LIHC (n = 370), LUAD (n = 504), PAAD (n = 177) and SARC (n = 259).

The prognostic value of ENO1 expression level in human cancers was analyzed using the Kaplan-Meier plotter database. We found that ENO1 upregulation was associated with poor overall survival (OS) in BLCA (n = 404, HR = 1.6, P = 0.0016; **Figure 2A**), BRCA (n = 1089, HR = 1.52, P = 0.015; **Figure 2B**), CESC (cervical squamous cell carcinoma) (n = 304, HR = 1.84, P = 0.0098; **Figure 2C**), ESCC (esophageal squamous cell carcinoma) (n = 81, HR = 2.34, P = 0.038; **Figure 2D**), HNSC (n = 499, HR = 1.35, P = 0.029; **Figure 2E**), LIHC (n = 370, HR = 2.29, P = 1.7e-06; **Figure 2F**), LUAD (n = 504, HR = 1.63, P = 0.0017; **Figure 2G**), PAAD (n = 177, HR = 1.7, P = 0.017; **Figure 2H**) and SARC (sarcoma) (n = 259, HR = 1.73, P = 0.0066; **Figure 2I**). In addition, patients with higher ENO1 expression level had poor relapse-free survival (RFS) in BRCA, LIHC, PAAD, SARC, and TGCT (testicular germ cell tumors). More details of ENO1-RFS relationships were analyzed by the Kaplan-Meier plotter database (**Supplementary Figure 1B–F** **)**. Concisely, overexpression of ENO1 was found associated with poor prognosis in multiple tumor types.

### 3.3 ENO1 knockout significantly reduced the growth and colony formation rate of PDAC cells

**Figure 3:**
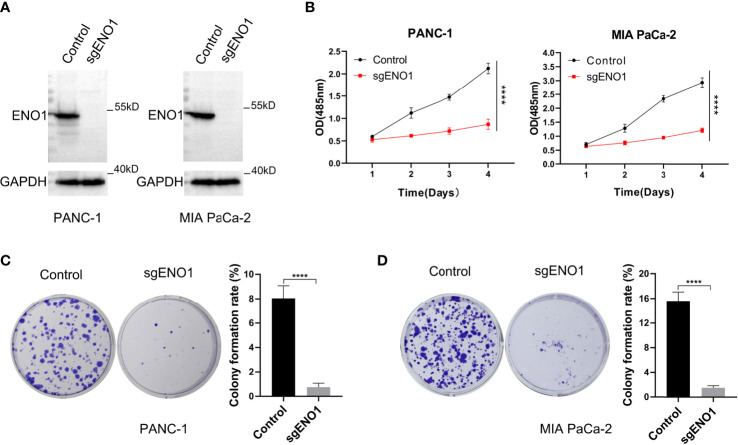
ENO1 knockout reduced the proliferation and colony formation ability of pancreatic cancer cells. **(A)** ENO1 knockout was validated by Western Blotting in PANC-1 and MIA PaCa-2 cells. **(B)** The cell proliferation (CCK-8 assay) and **(C, D)** colony formation rates were estimated in PANC-1 and MIA PaCa-2 cells after ENO1 knockout. 500 cells/well were seeded into the 6-well plates; ****P <0.0001.

To examine the biological function(s) of ENO1 in PC, we knocked out ENO1 in PANC-1 and MIA PaCa-2 cells **(** **Figure 3A** **)**. Both the ENO1-knockout and corresponding control cells were cultured and tested for cell viability using the CCK-8 assay. The cell growth curves (reflected by OD values) revealed that ENO1 knockout markedly inhibited the cell proliferation in both the PDAC cells **(** **Figure 3B** **)**. The cells were cultured for 10-15 days, the colonies were immobilized and stained, and then photographed and counted. We found that the colony numbers of the ENO1 knockout cells were sharply decreased compared to the corresponding control cells. ENO1 knockout decreased the proliferation abilities of PANC-1 and MIA PaCa-2 cells significantly compared with the control cells **(** **Figures 3C, D** **)**. Thus, knockout of ENO1 could inhibit the proliferation of PDAC cells.

### 3.4 ENO1 knockout decreased the cell migration, invasion, and glycolysis in PDAC cells

**Figure 4:**
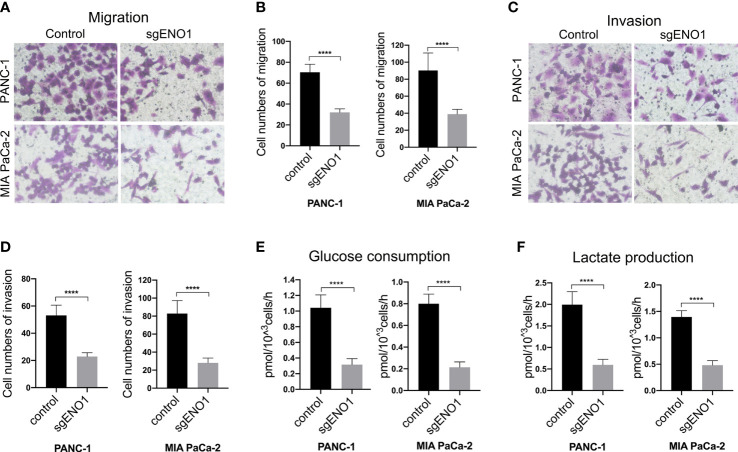
ENO1 knockout reduced cell migration, invasion, and glycolysis in PDAC cells. **(A, B)** The cell migration and **(C, D)** invasion decreased significantly after ENO1 knockout in PANC-1 and MIA PaCa-2 cells (transwell assay, ×200, n=3, error bars indicate S.D). **(E)** Glucose consumption and **(F)** lactate production significantly reduced after ENO1 knockout in PANC-1 and MIA PaCa-2 cells; ****P<0.0001.

The effect of ENO1 knockout on cell migration and invasion in PDAC cells was evaluated by transwell assay. We found that fewer cells migrated through the polycarbonate membrane in the ENO1 knockout groups than in the control groups **(** **Figures 4A, B** **)**. Consistently, when the polycarbonate membrane of the chamber was coated with Matrigel, the number of cells invading through the polycarbonate membrane was lower in the ENO1 knockout groups than in the control groups **(** **Figures 4C, D** **)**. These results revealed that ENO1 knockout significantly decreased the migration and invasion of PDAC cells. To examine the effect of ENO1 knockout on glycolysis, we detected the possible change in glucose and lactate concentrations in the two PDAC cells. The corresponding cell culture media were collected at 0 and 8 h to estimate the amounts of glucose and lactate, reflecting any possible change in glucose uptake and lactate secretion after ENO1 knockout. We found that the glucose consumption and lactate production in both the ENO1 knockout PDAC cells were significantly lower compared to that in corresponding control cells **(** **Figures 4E, F** **)**. This indicated that ENO1 knockout reduces glucose metabolism levels in PDAC cells.

### 3.5 ENO1 knockout inhibited the tumorigenicity of PDAC cells

**Figure 5:**
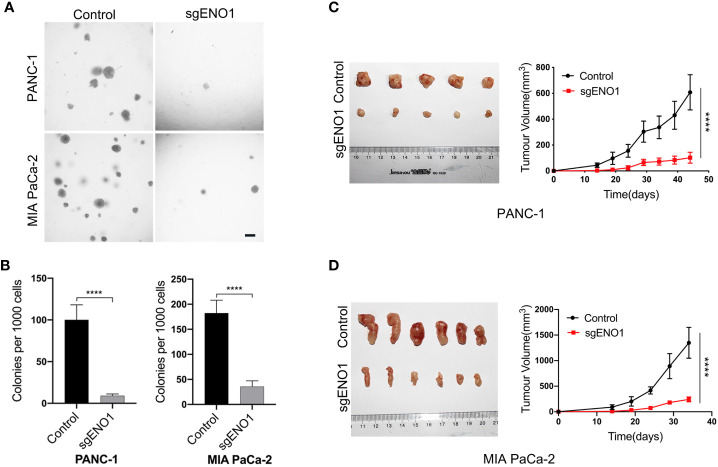
ENO1 knockout reduced tumorigenicity of PDAC cells. **(A, B)** The colony formation ability of PANC-1 and MIA PaCa-2 cells reduced after ENO1 knockout; scale bars, 100 μm. **(C, D)** The mice tumor growth curves originated from subcutaneous injection of ENO1 knockout and control cells are shown. (each group had 6 mice, one mouse died in the PANC-1 cell group); ****P<0.0001.

Cells were seeded into 24-well plates and cultured in 0.3% agar for 2-3 weeks. We found that the colony numbers were significantly lower in the ENO1 knockout groups than in the control groups **(** **Figures 5A, B** **).** This indicated that ENO1 knockout markedly reduced the tumorigenicity of PDAC cells *in vitro*. For *in vivo* assay, 2×10^6^ control or ENO1 knockout cells were subcutaneously inoculated into NOG mice. The tumor size was recorded every 5 days to obtain the growth curves. The results showed that tumor sizes were significantly smaller in ENO1 knockout mice than in control mice, indicating that ENO1 knockout reduced the tumorigenicity of PDAC cells **(** **Figures 5C, D** **)**.

### 3.6 ENO1 knockout induced compensatory upregulation of other metabolic pathways in PC

**Figure 6:**
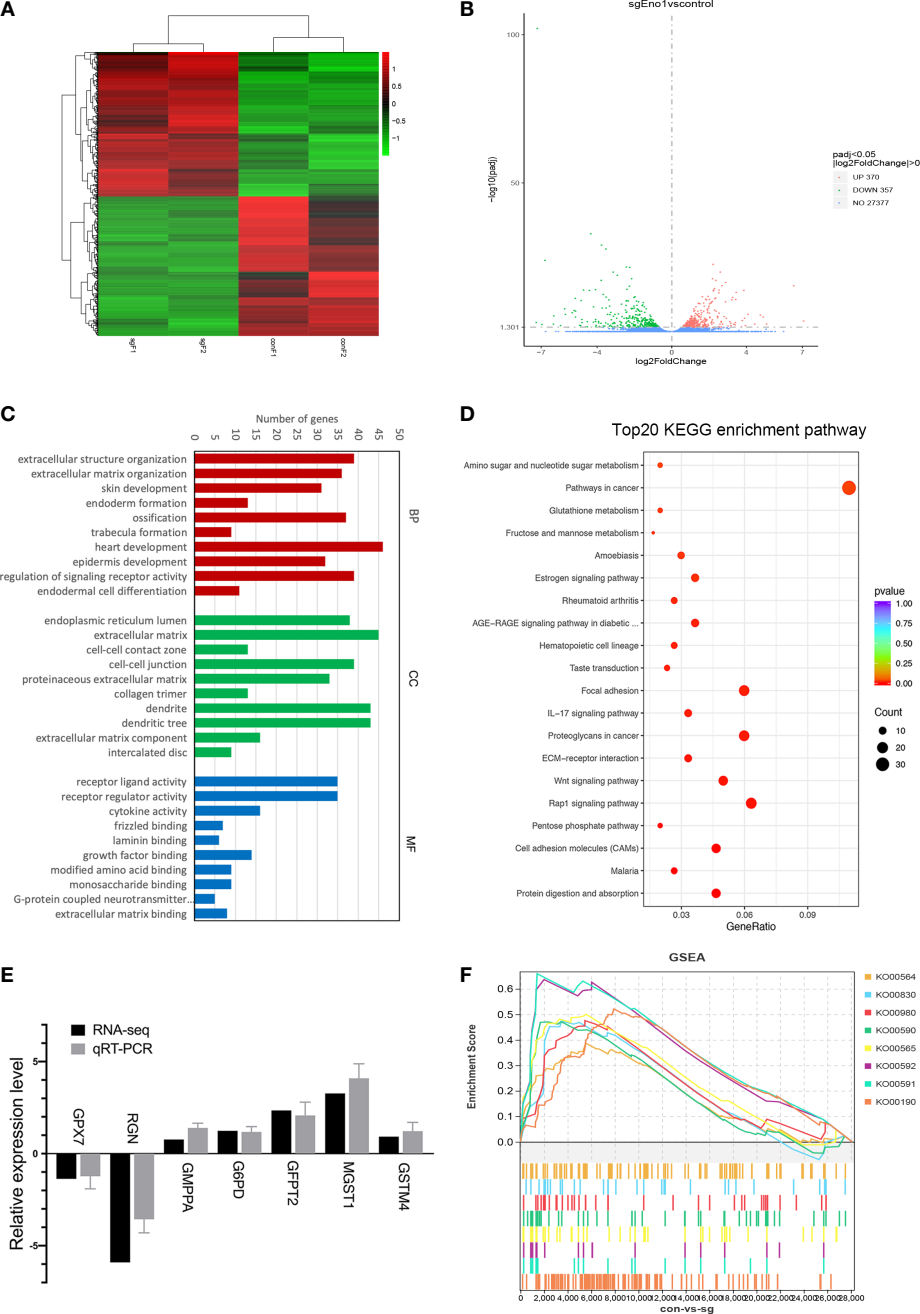
RNA-seq analysis of DEGs in control and ENO1 knockout groups. **(A)** A heatmap and **(B)** Volcano Plot of DEGs between ENO1 knockout and control cells: X-axis: the log2 (fold change); Y-axis: -log10 (Padj). Red and green dots represent the significantly upregulated and downregulated DEGs, respectively. **(C)** GO enrichment analysis of DEGs: X-axis, GO terms enriched in the BP, CC, and MF categories; Y-axis, the numbers of DEGs. **(D)** A scatterplot shows the DEGs enriched KEGG pathways: X-axis, the ratios of the DEGs involved in the pathways; Y-axis, the top 20 KEGG pathways based on -log_10_ (P-value). The bubble size and color respectively denote the number of DEGs and p-value of the corresponding KEGG pathways. **(E)**. qRT-PCR was used to validate the RNA-seq data; the X-axis shows the 7 randomly selected DEGs and Y-axis shows their relative expression levels. Grey and black columns represent the qRT-PCR and RNA-seq expression data, respectively. **(F)** GSEA plots illustrate the significant enrichment of metabolism-related upregulated genes in ENO1 knockout cells.

After examining the biological function of ENO1 in PC, we tried to understand its underlying molecular mechanism in abnormal cell metabolism in PC. Accordingly, RNA-seq was conducted to find the DEGs after ENO1 knockout in PANC-1 cells **(** **Supplementary Table 1** **)**. The heatmap from the transcriptome sequencing data revealed 727 DEGs (p-value <0.05), including 370 upregulated and 357 downregulated genes **(** **Figure 6A** **)**. These DEGs were also analyzed by a volcano map, which showed the overall changes in gene expression after ENO1 knockout. The red and green dots represent the upregulated and downregulated DEGs, respectively **(** **Figure 6B** **)**.

Furthermore, the identified DEGs were subjected to GO and KEGG pathway enrichment analysis. GO terms are defined as biological processes (BP), molecular functions (MF), and cellular components (CC). Based on the GO enrichment analysis, the top 20 GO terms of upregulated and downregulated genes were selected based on the -log_10_ (P-value) (**Supplementary Figures 2A–F** **)**. The top 10 GO terms indicated that the main BP terms enriching the DEGs were heart development and the regulation of signaling receptor activity; the enriched CC terms were extracellular matrix and dendrites; the enriched MF terms were receptor ligand and regulator activity **(** **Figure 6C** **)**. KEGG pathway analysis was used to annotate the DEGs related physiological and biochemical reaction pathways; the top 20 pathways were selected based on -log_10_ level (P-value). Many pathways showed substantial changes between the knockout and control cells **(** **Figure 6D** **)**. These pathways include the cancer pathways, Rap1 signaling pathway, Wnt signaling pathway, and many metabolic pathways. The number and percentages of the DEGs belonging to these pathways are listed in **Figure 6D**.

To understand how ENO1 participates in the abnormal metabolism of PDAC cells, we mainly focused on the identified cell metabolism-associated pathways, including the pentose phosphate pathway and metabolism of fructose, mannose, glutathione, amino, and nucleotide sugar. The DEGs involved in these four metabolic pathways and their relative expression levels (sgENO1 vs control) are listed in **Supplementary Table 2**. These DEGs may be the regulatory targets of ENO1. We randomly selected 7 DEGs to validate the RNA-seq data by qRT-PCR, including 5 upregulated and 2 downregulated genes. We found that RNA-seq data were consistent with qRT-PCR **(** **Figure 6E** **)**.

In addition, we performed GSEA to explore the potential downstream pathways of ENO1. GSEA highlighted that ENO1 knockout in PANC-1 cells positively associated with various genes related to oxidative phosphorylation (KO00190), ether lipid metabolism (KO00565), arachidonic acid metabolism (KO00590), linoleic acid metabolism (KO00591), alpha-linolenic acid metabolism (KO00592), retinol metabolism (KO00830), glycerophospholipid metabolism (KO00564), and metabolism of xenobiotics by cytochrome P450 (KO00980) **(** **Figure 6F** **)**. The GSEA plots, nominal p-value, FDR q-value, enrichment score (ES) and normalized ES nominated specifical metabolism pathways upregulated after ENO1 knockout are shown in **Supplementary Figures 3A–H**. The pathways were mainly associated with the activation of mitochondrial oxidative phosphorylation and lipid metabolism.

## 4 Discussion

Identifying the key regulators of PC is an important research challenge for the timely diagnosis and treatment of PC (19). In PC, tumor cells undergo reprogrammed metabolism to meet energy requirements and support malignant behaviors. Notably, dysregulation of ENO1 has been associated with several cancers (14, 20, 21). Multivariate analyses have shown that overexpression of ENO1 can be a predictor of tumor progression (11, 13). These studies suggest ENO1 is a promising therapeutic and diagnostic target in human tumors.

Here, we show that ENO1 is widely expressed in normal human tissues and is significantly upregulated in most TCGA (The Cancer Genome Atlas) cancer patients. Furthermore, OS analysis found that high expression of ENO1 was significantly associated with poor prognosis of BLCA, BRCA, CESC, ESCC, HNSC, LIHC, LUAD, PAAD, and SARC cancers. Concerning the RFS, high expression of ENO1 was significantly related to poor prognosis of BRCA, LIHC, SARC, PAAD, and TGCT cancers. These findings are consistent with prior studies showing a correlation of high ENO1 expression and poorer survival in cancer patients (22, 23). These data highlighted ENO1 as an oncogene in various cancers.

Recent study have shown that ENO1 overexpression promoted proliferation, invasion and migration of SKCM cells; and increased pyruvate and lactate production (24). Subsequently, we explored the hypothesis that ENO1 is one of the leading regulators of the Warburg effect and thus plays a major role in carcinogenesis and tumor maintenance. We found that ENO1 knockout markedly reduced the cell proliferation, migration, and invasion of PDAC cells; meanwhile, glycolysis levels and tumorigenicity were also reduced. RNA-seq analysis identified key 727 DEGs after ENO1 knockout, which are mainly distributed in the extracellular matrix and endoplasmic reticulum, and participate in the regulation of signal receptor activity. KEGG pathway enrichment analysis revealed that the DEGs were related to key cellular metabolism pathways, including the pentose phosphate pathway, and the metabolism of fructose, mannose, glutathione, and others. In addition, RAS oncogene family members RAP1B, RHOH and Wnt family member WNT9A expression level were significantly down-regulated in ENO1 knockout group. Interestingly, we found that the expression levels of stem cell marker CD24 and CD34 were significantly decreased after knockout of ENO1. This is consistent with the report that ENO1 regulates stem cell-like properties in cancer cells (25).

In this study, we found that ENO1 knockout in PDAC cells affected the expression of abundant metabolism-related genes, confirming that the inhibition of ENO1 decreases proliferation and also suppresses tumor growth *in vivo* and *in vitro*. G6PD in the pentose phosphate pathway was upregulated after ENO1 knockout, suggesting a shift of glucose metabolism to the pentose phosphate pathway in PDAC cells. It seems that ENO1 silencing can redistribute excessive glucose to the pentose phosphate pathway, decreasing lactate levels. Metabolic pattern changes can promote autophagy and fatty acid oxidation, reducing the growth of cancer cells (26). ENO1 knockout downregulated ALDOC levels in fructose and mannose metabolic pathways. The aberrant expression of aldolase family members has been demonstrated to promote tumor progression; ALDOC is upregulated in various cancers and acts as a regulator of Wnt signaling (27). The knockdown of ALDOC reduces cell growth, glucose uptake, and glycolysis in cancer cells (28). Silencing of UAP1 inhibited the growth and colony formation of cancer cells (29). Our RNA-seq data showed that UAP1 levels decreased affecting the amino and nucleotide sugar metabolism pathway. These genes may be indispensable to ENO1-mediated regulation of cell metabolism in PC, however, the biological functions of other DEGs in PC must be examined in the future. Meanwhile, GSEA revealed that genes encoding for mitochondrial functions and lipid metabolism were significantly upregulated in ENO1 knockout cells, indicating a shift in metabolism patterns in cancer cells.

In summary, our findings show the metabolic analysis following ENO1 knockout. ENO1 is a potential oncogene and its knockout may suppress the tumorigenicity of PDAC cells by triggering the metabolic pattern change from glycolysis to other metabolic pathways such as pentose phosphate pathway, mitochondrial OXPHOS and lipid metabolism. ENO1 can be exploited as a therapeutical target for reducing aerobic glycolysis in PC. Inhibition of ENO1 alone or in combination with other pathways activated by ENO1 knockout, opens novel avenues for future cancer therapeutic approaches. Nonetheless, this study had certain limitations. We mainly validated the biological functions of ENO1 in PDAC cells by examining “loss-of-function”, while “gain-of-function” studies can further uncover the mechanism of ENO1 activating intracellular and extracellular signals in cancer cells.

## Data availability statement

The datasets presented in this study can be found in online repositories. The names of the repository/repositories and accession number(s) can be found below: The raw sequencing data have been deposited in the Genome Sequence Archive (GSA) database under accession number HRA001089. All of the data are also available as the sequence read archive (SRA) format in the National Center for Biotechnology Information (NCBI) with the accession number of SRP414959.

## Ethics statement

The animal study was reviewed and approved by Peking University Cancer Hospital Animal Care and Use Committee.

## Author contributions

ZZ, QS, and KZ designed the study. QS and KZ did experiments. QS, KZ, TS, and CX finished computational analysis. WZ provided expert consultation. ZZ, QS, and KZ wrote the manuscript. ZZ supervised the study. All authors contributed to the article and approved the submitted version.
