# Supplementary material for: Knockout of ENO1 leads to metabolism reprogramming and tumor retardation in pancreatic cancer
Source: Front Oncol. 2023 Feb 10;13:1119886. doi: 10.3389/fonc.2023.1119886 (PMC9950624; doi:10.3389/fonc.2023.1119886)
Supplement: Supplementary file 1 [file DataSheet_1.docx]

**Supplementary Figure1**

**
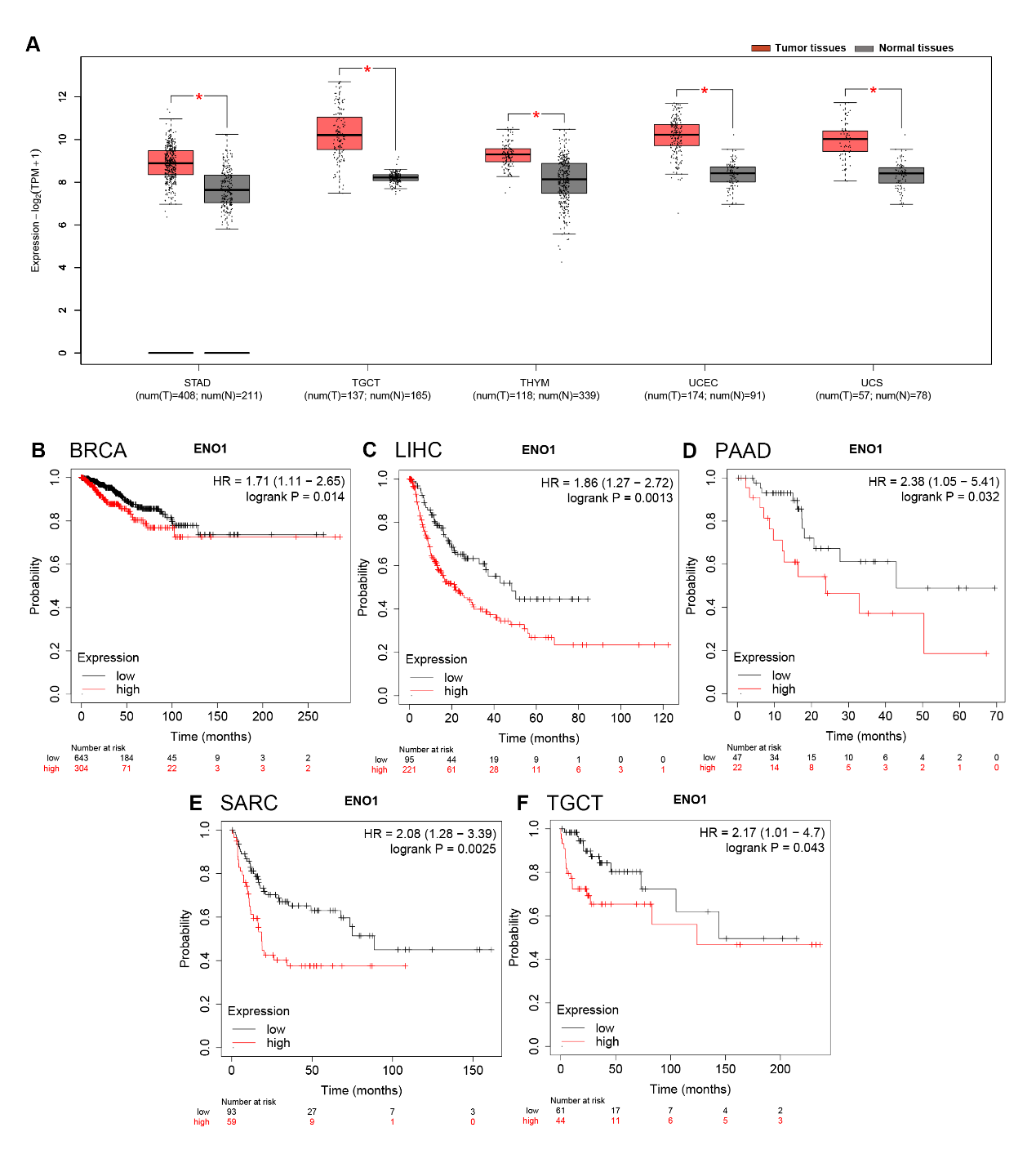
**

**Supplementary Figure1.** **The relationship between ENO1 expression and different cancers from online database.**  (A) The expression of ENO1 in different cancer tissue and paired normal tissue analyzed by the GEPIA database. (B-F) Kaplan-Meier relapse-free survival curve of human cancers with high and low ENO1 expression analyzed by the Kaplan-Meier plotter database. RFS, relapse-free survival.

**Supplementary Figure2**

**
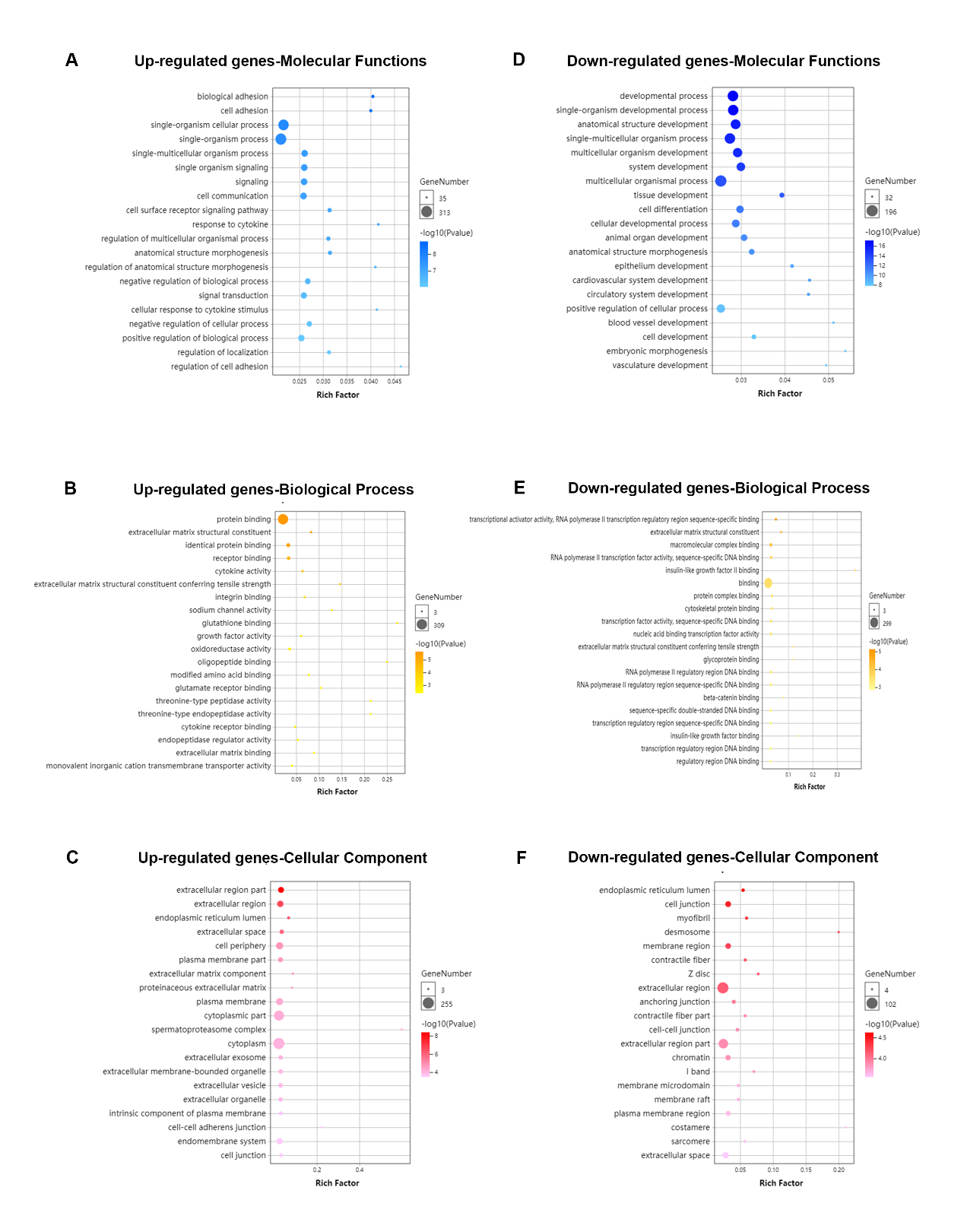
**

**Supplementary Figure2. GO enrichment analysis of up-regulated (P<0.05, Log2 (Fold change)>1) and down-regulated genes (P<0.05, Log2 (Fold change) <-1) based on the ENO1 knockout groups and control groups.**

**Supplementary Figure3**


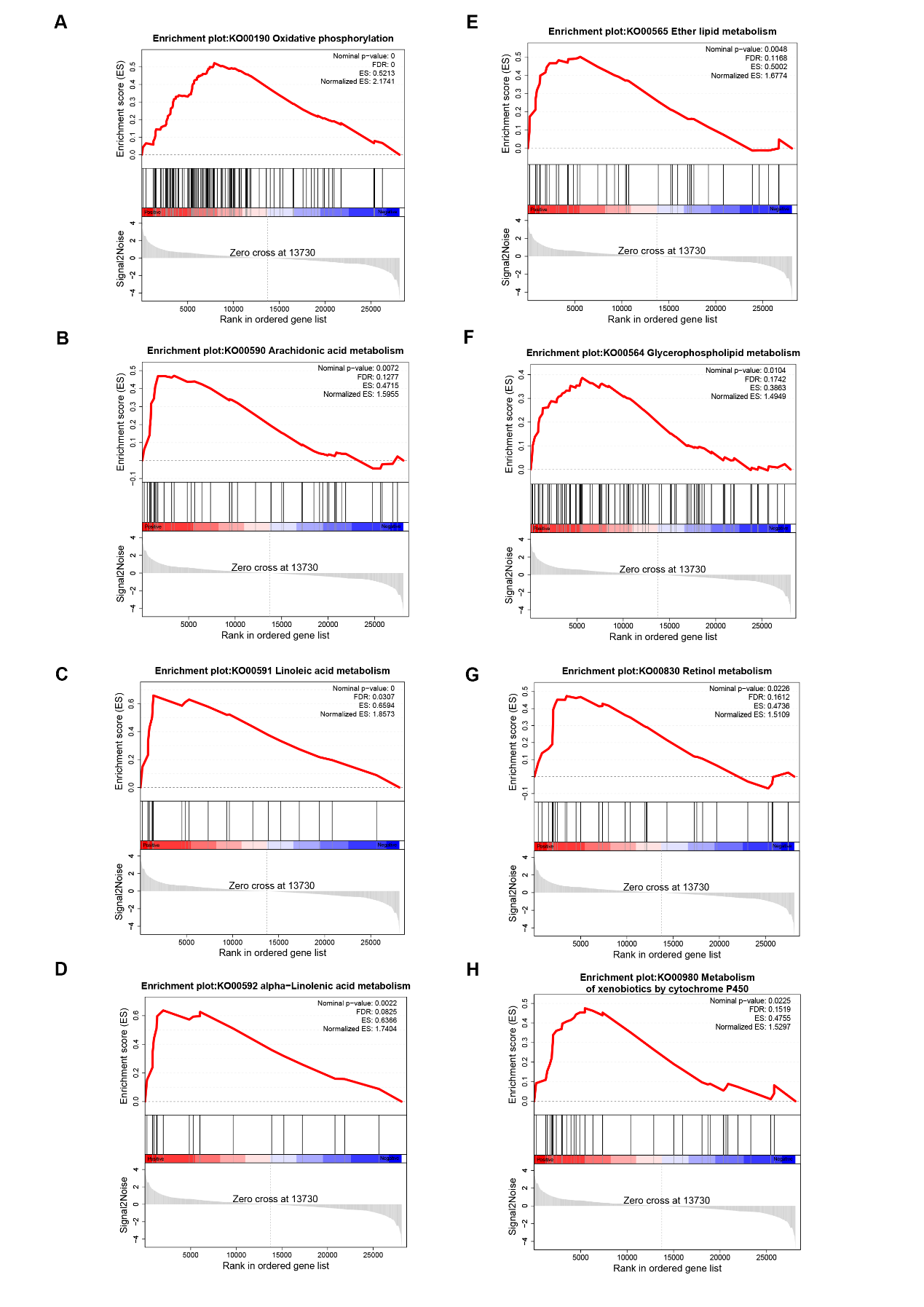


**Supplementary Figure3. GSEA plot focused on specifical gene sets involving metabolism pathways showed significant enrichment in PANC-1 cells expressing**

**ENO1 KO.**
