# Supplementary material for: Knockout of ENO1 leads to metabolism reprogramming and tumor retardation in pancreatic cancer
Source: Front Oncol. 2023 Feb 10;13:1119886. doi: 10.3389/fonc.2023.1119886 (PMC9950624; doi:10.3389/fonc.2023.1119886)
Supplement: Supplementary file 2 [file DataSheet_2.docx]

figures data: https://www.jianguoyun.com/p/Dd56FJYQsraeCxil8usEIAA
