# Supplementary material for: Knockout of ENO1 leads to metabolism reprogramming and tumor retardation in pancreatic cancer
Source: Front Oncol. 2023 Feb 10;13:1119886. doi: 10.3389/fonc.2023.1119886 (PMC9950624; doi:10.3389/fonc.2023.1119886)
Supplement: Supplementary file 4 [file Table_2.docx]

**Supplementary Table 2**

The relative expression level of DEGs involved in the KEGG pathways

| Genes | Log_2_ (fold change) | Corrected P-value |
| --- | --- | --- |
| Pentose phosphate pathway  G6PD  ALDOC  RGN  PGD  PFKM  GLYCTK | 1.24  -1.11  -5.91  0.71  0.78  -1.60 | 1.45E-07  0.01015817  0.01282931  0.02106969  0.02197448  0.04681251 |
| Fructose and mannose metabolism  AKR1B1  HK1  ALDOC  GMPPA  PFKM | 1.11  -0.81  -1.11  0.77  0.78 | 0.00035423  0.00202984  0.01015817  0.01171708  0.02197448 |
| Glutathione metabolism  MGST1  G6PD  GPX7  PGD  GPX8  GSTM4 | 3.27  1.24  -1.37  0.71  0.98  0.92 | 2.28E-11  1.45E-07  0.00792186  0.02106969  0.02522728  0.04945702 |
| Amino sugar and nucleotide sugar metabolism |  |  |
| GFPT2 | 2.35 | 5.93E-08 |
| HK1  GMPPA  UGDH  GALE | -0.81  0.76  -0.72  0.71 | 0.00202984  0.01171708  0.01422217  0.01733457 |
| UAP1 | -0.80 | 0.0412074 |
